# Lys-315 at the Interfaces of Diagonal Subunits of δ-Crystallin Plays a Critical Role in the Reversibility of Folding and Subunit Assembly

**DOI:** 10.1371/journal.pone.0145957

**Published:** 2016-01-05

**Authors:** Chih-Wei Huang, Hui-Chen Lin, Chi-Yuan Chou, Wei-Chuo Kao, Wei-Yuan Chou, Hwei-Jen Lee

**Affiliations:** 1 Department of Biochemistry, National Defense Medical Center, Taipei, Taiwan; 2 Pharmacy Division, Kaohsiung Armed Forces General Hospital, Kaohsiung, Taiwan; 3 Graduate Institute of Medical Sciences, National Defense Medical Center, Taipei, Taiwan; 4 Department of Life Sciences and Institute of Genome Sciences, National Yang-Ming University, Taipei, Taiwan; Jamia Millia Islamia, INDIA

## Abstract

δ-Crystallin is the major structural protein in avian eye lenses and is homologous to the urea cycle enzyme argininosuccinate lyase. This protein is structurally assembled as double dimers. Lys-315 is the only residue which is arranged symmetrically at the diagonal subunit interfaces to interact with each other. This study found that wild-type protein had both dimers and monomers present in 2–4 M urea whilst only monomers of the K315A mutant were observed under the same conditions, as judged by sedimentation velocity analysis. The assembly of monomeric K315A mutant was reversible in contrast to wild-type protein. Molecular dynamics simulations showed that the dissociation of primary dimers is prior to the diagonal dimers in wild-type protein. These results suggest the critical role of Lys-315 in stabilization of the diagonal dimer structure. Guanidinium hydrochloride (GdmCl) denatured wild-type or K315A mutant protein did not fold into functional protein. However, the urea dissociated monomers of K315A mutant protein in GdmCl were reversible folding through a multiple steps mechanism as measured by tryptophan and ANS fluorescence. Two partly unfolded intermediates were detected in the pathway. Refolding of the intermediates resulted in a conformation with greater amounts of hydrophobic regions exposed which was prone to the formation of protein aggregates. The formation of aggregates was not prevented by the addition of α-crystallin. These results highlight that the conformational status of the monomers is critical for determining whether reversible oligomerization or aggregate formation occurs.

## Introduction

δ-Crystallin is a taxon-specific eye lens protein. It is the major soluble protein in the eye lens of reptiles and birds and functions as a structural protein to maintain the refraction properties of the lens [1,2]. δ-Crystallin and argininosuccinate lyase (ASL) are homologous proteins. ASL is responsible for the conversion of argininosuccinate into arginine and fumarate in the urea cycle. δ-Crystallin and ASL share about 70% amino acid sequence identity and function as homotetramers, with four identical multi-subunit active sites [1–6].

**Fig 1:**
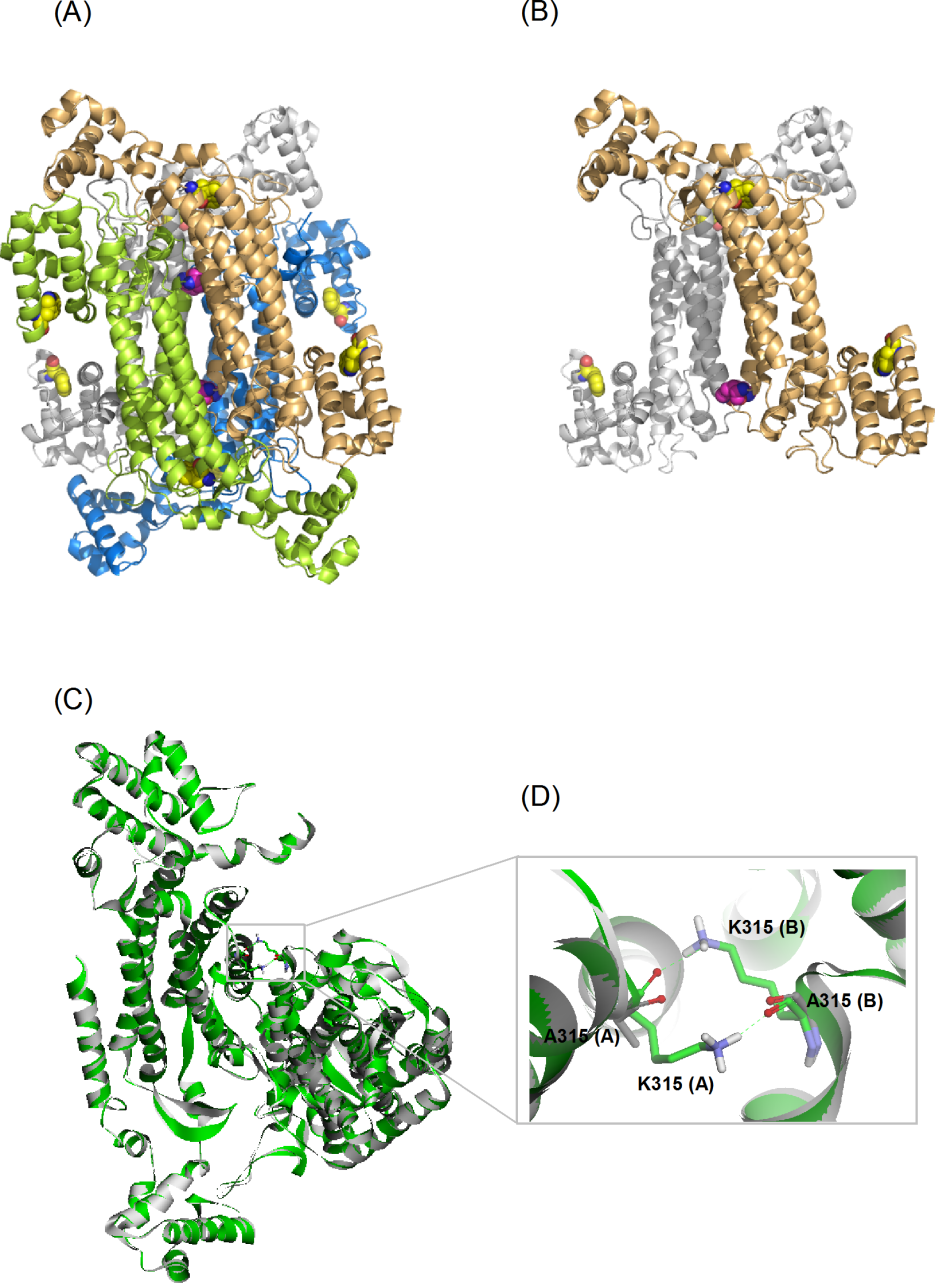
The structure of goose δ–crystallin. (A) The quaternary structure (PDB accession no: 1XWO) and (B) the A-B dimeric pairs from diagonal subunits. Monomers of A, B, C and D are colored as green, blue, yellow and gray, respectively. Residues of K315 and W74/W169 are highlighted as CPK and colored as magenta and yellow, respectively. (C) Superimposition of wild-type and K315A mutant proteins present as green and grey color, respectively. A expand view of the interactions of K315 at the interface of A and B subunit is highlighted in (D). Residues of K315 and A315 are displayed as stick models. The interactions are present by dashed green lines.

δ-Crystallin and ASL have similar X-ray crystal structures. Each monomer consists of three domains. The helices in domain 2 of each monomer associate to form a central helix bundle, comprising the core structure of the protein (Fig 1A) [4,5,7–10]. The active sites of the enzyme are located in a cleft between three different monomers [4]. The quaternary structure of δ-crystallin consists of a double dimer. The contact surface between the two dimers is smaller than the interface within the primary dimer of the structure [11]. Hydrogen bonding, salt bridges and hydrophobic interactions are the major forces which stabilize the quaternary structure of the protein.

In the presence of guanidinium chloride (GdmCl), tetrameric δ-crystallin is unfolded *via* a multistep pathway involving subunit dissociation into a monomeric molten globule intermediate, followed by denaturation [12,13]. The dimeric form is transiently detected during this unfolding/refolding process. These dimers are unstable and they are prone to self-associate into protein aggregates, and this process competes with the formation of native tetramers [8]. Hence, the assembly of two dimers acts as a kinetic barrier in the refolding pathway [14]. The proper assembly of double dimers is thus important for producing a stable δ-crystallin quaternary structure.

The N-terminus of δ-crystallin has been identified as being critical for the proper assembly of the double dimers [8]. In the quaternary structure, the first 25 N-terminal amino acid residues protrude into the neighboring monomer and interact with a hydrophobic cavity. When this sequence of amino acids was deleted the protein became unstable and was prone to form protein aggregates. The salt bridge formed by residues of R302 and E330, located in the helix bundle at the dimer-dimer interface, is also important interaction for stabilization of the quaternary structure of δ-crystallin. When this interaction was disrupted by site-directed mutagenesis, the exchange rate of subunits was dramatically accelerated [15]. The interactions of E327 with both K299 and R302 and the interaction of E267 with Y158 at the dimer interface were found to stabilize the quaternary structure of δ-crystallin in a cooperative manner. Mutations of the residues involved in both these interactions caused the majority of dimers to dissociate, whilst only partial dissociation was observed when these interactions were individually disrupted, as judged by sedimentation velocity experiments [11].

Inspection of the structure of δ-crystallin showed that the primary interactions between two symmetrically associated monomers in diagonal positions were provided by residues located at the top and bottom sides of the helical bundles (Fig 1B). K315 is one of the residues symmetrical located at this interface, interacting with the same residue in other monomers by hydrogen bonds (Fig 1C and 1D). Substitution of this residue with leucine resulted in part dissociation of the quaternary structure. In contrast, the K315A mutant protein was relatively stable. This protein was unfolded into an intermediate with a stable conformation at 3 M urea [11]. This phenomenon was not observed for the K315L mutant protein, which was unfolded under the same conditions. The results lead to the hypothesis that the bulky and charged side-chain of K315 might affect the stability of the intermediate during the process of protein unfolding. When wild-type δ-crystallin was unfolded in GdmCl, the first transition for subunit dissociation resulted in a monomeric intermediate with a molten globule conformation [12,13]. During the refolding of 5 M GdmCl denatured δ-crystallin a marked hysteresis was observed, suggesting that the quaternary structure of that inappropriate assembled species might be related to the conformation of the refolded monomers [14,16]. The presence of stable intermediate during unfolding of K315A mutant protein in the presence of urea suggests that the interactions provided by this residue at the interfaces might provide an energy barrier for subunit dissociation. In this study, the effects of this interaction on the folding pathway of wild-type and mutant proteins were investigated using urea as a denaturant. The different distributions of dissociated component from wild-type and mutant proteins, as measured by sedimentation velocity experiment, suggests the quaternary structure dissociates in different ways for wild-type and mutant proteins. Structural simulation supports different dissociation processes for the two proteins. These results highlight the critical role of K315 in stabilizing the quaternary structure of δ-crystallin. The residue appears to control both the dissociation of dimers into monomers and the stability of the produced monomers. The monomers dissociated from the K315A mutant protein with a stable and compact conformation provided a good model for studying the folding mechanism of the δ-crystallin. This study reveals the conformational status of the monomers, which determines whether functional protein or aggregates are formed.

## Materials and Methods

### Protein production and purification

The recombinant wild-type and the K315A mutant δ-crystallin or αA-crystallin plasmid were transformed and over-expressed in *E*. *coli* BL21 (DE3) with induction by isopropyl-β-D-thiogalactopyranoside (IPTG). Proteins were purified as previously described [8,17]. The supernatants of crude cell extracts were loaded onto a Q-Sepharose anion exchange column (HiPrep 16/10 Q XL, GE Healthcare) pre-equilibrated in buffer A (50 mM Tris-HCl buffer, pH 7.5) and eluted with a linear gradient of 0 to 0.4 M NaCl in buffer A. Recombinant protein was eluted at approximately 0.15 M NaCl. The eluted protein was pooled and treated with ammonium sulfate to 1.2 M. After filtration, the sample was loaded onto a hydrophobic interaction column (Source™ 15PHE) pre-equilibrated in buffer A containing 10% (v/v) glycerol and 1.2 M ammonium sulfate and eluted with a linear gradient to the same buffer lacking ammonium sulfate. The retained proteins were eluted at ~0.3 M ammonium sulfate. Fractions were pooled and loaded onto S-300 Sephacryl column (26 mm x 85 cm) pre-equilibrated in 50 mM Tris-acetic acid buffer, pH 7.5. Fractions were analyzed by SDS-PAGE and protein concentrations determined by the method of Bradford [18]. Proteins possessing a C-terminal His_6_ tag were purified on Ni affinity column (Chelating Sepharose FF, GE Healthcare) then desalted using a Sephadex G-25 column (26 mm x 12 cm) as previously reported [11].

### Equilibrium unfolding and refolding experiments

Equilibrium unfolding experiments were carried out by overnight incubation of δ-crystallin with various concentrations of urea or GdmCl in 50 mM Tris-acetic acid buffer, pH 7.5 at 25°C. The refolding experiments were undertaken by dilution of equilibrium-denatured δ-crystallin to a series of urea or GdmCl concentrations in the same buffer.

The experiments for equilibrium unfolding of monomeric δ-crystallin were undertaken by overnight incubation of δ-crystallin in 1.5 M urea at 25°C followed by addition of various concentrations of GdmCl to the solution followed by incubation for 2 hrs. The refolding experiments were carried out by dilution of the denatured δ-crystallin into a solution containing 1.5 M urea and 0.8, 3 or 5 M GdmCl in 50 mM Tris-acetic acid buffer, pH 7.5. To analyze the conformation and quaternary structure of the refolded δ-crystallin, the refolding experiments were undertaken by 20-fold dilution of the denatured monomeric δ-crystallin with buffer.

### Data analysis

**Fig 2:**
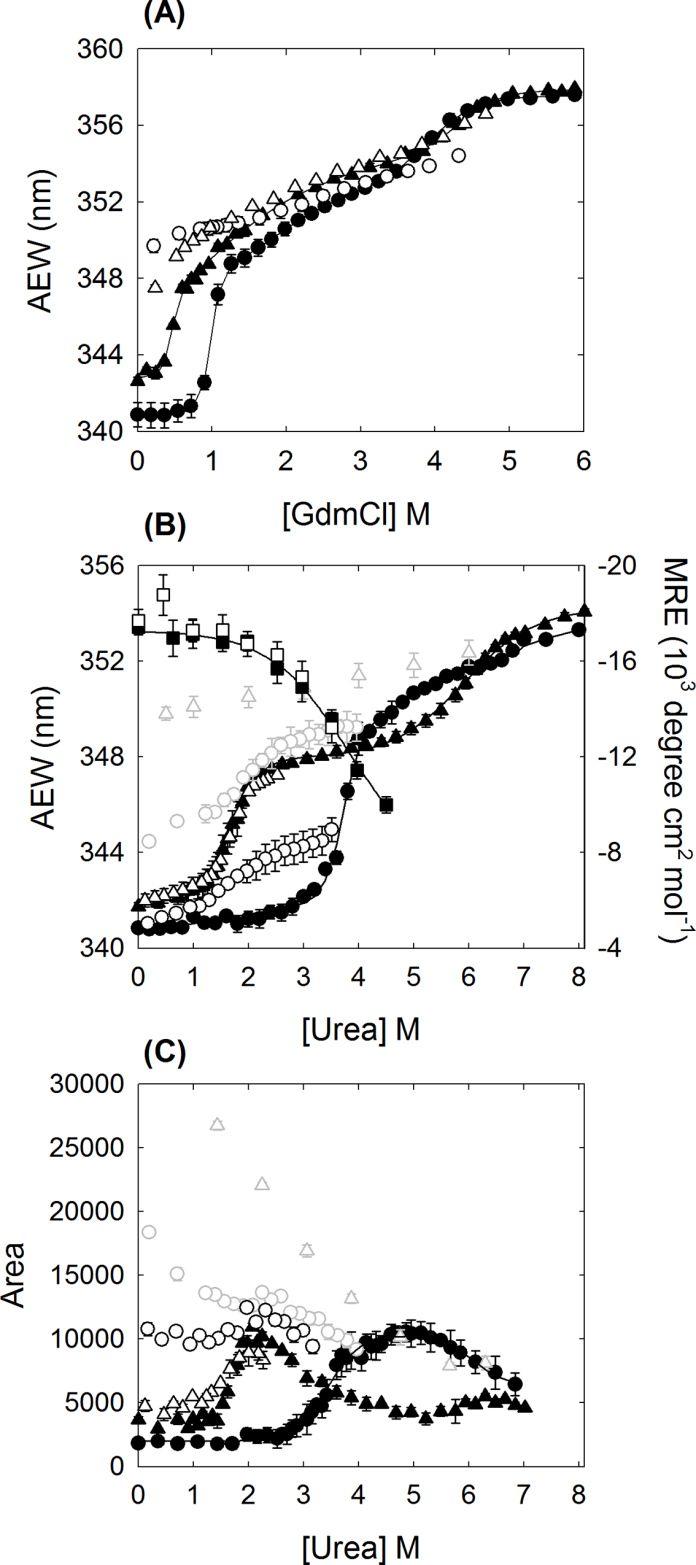
The unfolding transition of the wild-type and mutant δ-crystallin. Changes in average emission wavelength (AEW) of tryptophan fluorescence are represented as a function of GdmCl (A) and urea (B) concentration. (C) The integrated area of the fluorescence emission spectrum of ANS at varied urea concentration. The labels of (●) and (○), and (▲) and (∆) represent the unfolding and refolding of wild-type and K315A mutant protein, respectively. In (B) and (C), the dark or grey open circles and triangles represent the refolding transition from 3.5 or 4 M urea denatured wild-type and 2.5 or 6 M urea denatured mutant protein, respectively. The (■) and (□) in (B) represent the changes of mean residue of ellipticity (MRE) in the unfolding of wild-type and mutant protein, respectively. The protein concentrations used in the assays were 0.03, 0.2 and 0.1 mg/mL for tryptophan fluorescence, CD and ANS fluorescence measurements, respectively.

The transition region in Fig 2B for the reversible dissociation of K315A mutant δ-crystallin in urea solution was analyzed using the following method. The unfolding process was described as a two-state transition for the conversion of the tetramer (T) into monomers (M). The thermodynamic parameters were obtained by global fitting of the tryptophan fluorescence signal to Eq 1 [19]:

$$y_{0}=\{(y_{\text{n}}+m_{\text{f}}[\text{D}])+(y_{\text{u}}+m_{\text{u}}[\text{D}])\text{exp}(-(\Delta G_{\text{u}}{}^{0}\;-\;m[\text{D}])/RT)\}/\{1+\text{exp}(-(\Delta G_{\text{u}}{}^{0}\;-m[\text{D}])/RT)\}$$ (1)

where *y* is the observed signal from tryptophan fluorescence, *y*_n_ and *y*_u_ are the signals in the folded and unfolded states, and *m*_f_ and *m*_u_ are the slopes of the baselines preceding and following the transition region. Δ*G*_u_^0^ is the free energy difference in the absence of urea and *m* is the variation in the free energy of unfolding with the urea concentration.

Reversible unfolding of monomeric K315A mutant δ-crystallin were described as a four state process (M↔I_1_↔I_2_↔U). The unfolding curve from tryptophan fluorescence was analyzed using a four-state unfolding model described as the transition from N to U with two intermediates, I_1_ and I_2_, in the process. The thermodynamic parameters were calculated by fitting the data to Eq 2 [8,20]:

$$\begin{array}{l} y_{0}=\{y_{\text{N}}+y_{\text{I}1}\;\text{exp}\{-(\Delta G_{1}{}^{0}-m_{1}[\text{D}])\;/\;RT\}+y_{\text{I}2}\;\text{exp}\{-(\Delta G_{1}{}^{0}-m_{1}[\text{D}]+\Delta G_{2}{}^{0}-m_{2}[\text{D}])\;/\;RT\}+\\ y_{\text{U}}\;\text{exp}\{-(\Delta G_{1}{}^{0}-m_{1}[\text{D}]+\Delta G_{2}{}^{0}-m_{2}[\text{D}]+\Delta G_{3}{}^{0}-m_{3}[\text{D}])\;/\;RT\}\}\;/\;\{1+\text{exp}\{-(\Delta G_{1}{}^{0}-m_{1}[\text{D}])\;/\;RT\}\\ +\text{exp}\{-(\Delta G_{1}{}^{0}-m_{1}[\text{D}]+\Delta G_{2}{}^{{}_{0}}-m_{2}[\text{D}])\;/\;RT\}+\text{exp}\{-(\Delta G_{1}{}^{0}-m_{1}[\text{D}]+\Delta G_{2}{}^{0}-m_{2}[\text{D}]+\Delta G_{3}{}^{0}-\\ m_{3}[\text{D}])\;/\;RT\}\} \end{array}$$ (2)

where *y*_I1_ and *y*_I2_ are the signals in the I_1_ and I_2_ states. Δ*G*_1_^0^, Δ*G*_2_^0^ and Δ*G*_3_^0^ are the free energy differences in the absence of GdmCl for N to I_1_, I_1_ to I_2_ and I_2_ to U transition, respectively, and *m*_*1*_, *m*_*2*_ and *m*_*3*_ are the variation in the free energy of unfolding with the GdmCl concentration.

### Enzymatic activity assay

δ-crystallin was assayed for ASL activity by monitoring the absorption of fumarate at 240 nm in a Perkin-Elmer Lambda 40 spectrophotometer. Assays were performed at least in triplicate in 50 mM Tris-HCl buffer (pH 7.5) with 1 mM sodium argininosuccinate as substrate. A molar absorption coefficient of 2.44 x 10^3^ M^-1^cm^-1^ was used for all calculations [21].

### Fluorescence studies

The fluorescence spectra were measured on a Perkin-Elmer LS-50 luminescence spectrophotometer at 25°C. Intrinsic tryptophan fluorescence spectra of the protein were recorded with excitation wavelength set at 295 nm and using 5 nm band-width for both excitation and emission wavelength. All spectra were corrected for buffer or denaturant absorption. The average emission wavelength was utilized for data analysis [22].

The ANS (1-anilinonaphthalene-8-sulfonic acid) (Molecular Probes; Eugene, Oregon) was used as probe to bind with proteins and the fluorescence was recorded from 450 to 550 nm with the excitation wavelength set at 370 nm. The band-widths were set at 5 nm for both the excitation and emission wavelengths. The final concentration of ANS was 0.2 mM.

### Circular dichroism studies

The circular dichroism (CD) spectra were measured on a Jasco J-810 spectropolarimeter at 25°C. Experiments were performed in 20 mM Tris-acetic acid buffer (pH 7.5) with a 1 mm path-length over a wavelength range from 200 to 250 nm. All spectra were averaged from three accumulations and were buffer corrected. The observed ellipticity (θ) (degrees) was converted into the mean residue ellipticity [θ] by the equation: [Θ] = Θ×M_MRW_/10×*d*×*c* [23], where M_MRW_ is the mean residue weight, *d* is the light path (cm), and *c* is the concentration of protein in g/ml.

### Analytical ultracentrifugation studies

The protein sedimentation were performed on a Beckman-Coulter (Palo Alto, CA) XL-A analytical ultracentrifuge (AUC) with an An50 Ti rotor at 20°C with 130 000 *g* in standard double sectors aluminum centerpieces. The radial scans were recorded with a time interval of 7-min and a step size of 0.003 cm. All data were fitted to the continuous c(s) distribution model and a continuous size-distribution with respect to frictional ratio (*f/f*_o_) model using the SEDFIT program [24,25]. The partial specific volume of the protein, solvent density and viscosity were calculated using the SEDNTERP program [26]. The solvent density and viscosity of varied urea concentrations were calculated and included in the fitting.

### Non-denaturing gradient gel electrophoresis

Electrophoresis was performed using a PhastSystem (GE Healthcare). The samples were subjected a PhastGel 4–15% gradient gel which contains the Native Buffer Strip (0.88 M L-alanine, 0.25 M Tris/HCl pH 8.8) attached to the surface of the gel and both electrodes. The electrophoresis was carried out at 10 mA and 15^***°***^C for 400 Vh. After electrophoresis, the gel was fixed in 20% (w/v) trichloroacetic acid and stained with Coomassie Brilliant Blue R250.

### Protein aggregation measurements

The turbidity of protein solution was measured using a PerkinElmer Lambda 40 spectrophotometer equipped with a Peltier temperature control accessory to monitor the light scattering at 360 nm. All measurements were carried out at 25°C. Spectra were corrected using measurements recorded for native protein in the absence of denaturants. The aggregation rate was calculated by fitting the data to the single or double exponential equation:

$$y_{\text{t}}=y_{0}+\sum_{i=1}^{n}y_{\text{i}}\left[1-\text{exp}\left(-k_{\text{i}}t\right)\right]$$

Where *y*_t_ is the signal at time t, *y*_0_ is the signal of the final state, *y*_i_ is the change in amplitude, and *k*_i_ is the rate constant for aggregation. Data for the linear increase in signals was fitted to a linear equation using SigmaPlot 10.

### Thioflavin T assay

The assay was performed by setting the excitation wavelength at 440 nm and measuring the emission spectrum from 460 to 600 nm. Proteins unfolded in denaturant were diluted into 50 mM Tris-acetic acid buffer (pH 7.5) to give 0.05 mg/ml of protein in the presence of thioflavin T (ThT) (50 μM). The spectrum of ThT alone was used as a correction for each assay.

### Structural simulation

The crystal structure of goose δ–crystallin (PDB code: 1XWO) with all water molecule removed was subjected to the CHARMm force field and energy minimized with the smart minimization algorithm to satisfy (RMS Gradient ~0.1 kcal/mol/Å). The implicit solvent model of Generalized Born was included in the calculation. The structural model was used as a template to build the K315A mutant model using the build mutant protocol (Accelrys Discovery Studio 3.5, Accelrys Inc.). The best scoring model conformation was selected for energy minimization.

Molecular Dynamics (MD) simulations were performed using the standard dynamics cascade protocol. The structures of wild-type and mutant δ-crystallin in the CHARMm force field were subjected to initial energy minimization for 500 steps by steepest descent followed by a conjugate gradient for 500 steps. The minimized models were then heated from 50 to 300 K in 2 ps MD simulations followed by equilibration for 2 ps at 300 K in the absence of any structural restraint. Finally, the equilibrated models were submitted to MD simulations for 100 ps at NVT (constant number of particles, volume, and temperature) using the Berendsen coupling algorithm [27].

## Results

### Conformational reversibility of δ-crystallins in the presence of denaturants

Wild-type and K315A mutant δ-crystallin purified to near homogeneity were used for all analysis. Equilibrium unfolding experiments were conducted by incubation of proteins in buffer supplemented with different GdmCl or urea concentrations overnight. Tryptophan fluorescence was used to monitor the conformational changes during the unfolding process in the microenvironment around the tryptophan residues (Fig 2A and 2B). There are two tryptophan residues, W74 and W169, in the structure of δ-crystallin. They are located in the solvent accessible domain 1 and the helix bundle of domain 2, respectively (Fig 1A and 1B). Unfolding of the wild-type and K315A mutant protein follows a multistep process in GdmCl solution and was not reversible after 20-fold dilution (Fig 2A). As shown in Fig 2A, the dramatic changes in the signal for the first transition were due to subunit dissociation as reported previously, with the GdmCl concentrations for half transition ([D]_1/2_) at 1 ± 0.05 and 0.5 ± 0.01 M for wild-type and K315A mutant protein, respectively [28]. Unfolding of the two proteins in urea solution followed a three-state process, with the [D]_1/2_ values in the first transition at 3.6 ± 0.1 and 1.7 ± 0.1 M urea, respectively (Fig 2B) [8,12]. The differences in the denaturant concentration required for the half transition clearly show the potency of GdmCl when disrupting of the conformation of δ-crystallin [11].

In the presence of urea, the K315A mutant protein was dissociated and stayed in a stable conformation between 2 and 5 M urea. The conformation was more stable than for wild-type protein at urea concentrations exceeding 4 M. When the urea was removed by 20-fold dilution into buffer, the denatured mutant protein was able to refold into a conformation similar to the original state (Fig 2B). In contrast, dilution of 3.5 or 4 M urea denatured wild-type δ-crystallin or 6 M urea denatured K315A mutant protein did not result in the restoration of the properly folded conformation. These results suggest that the reversible assembly of the quaternary structure of δ-crystallin is correlated with the conformation of the dissociated monomers.

**Fig 3:**
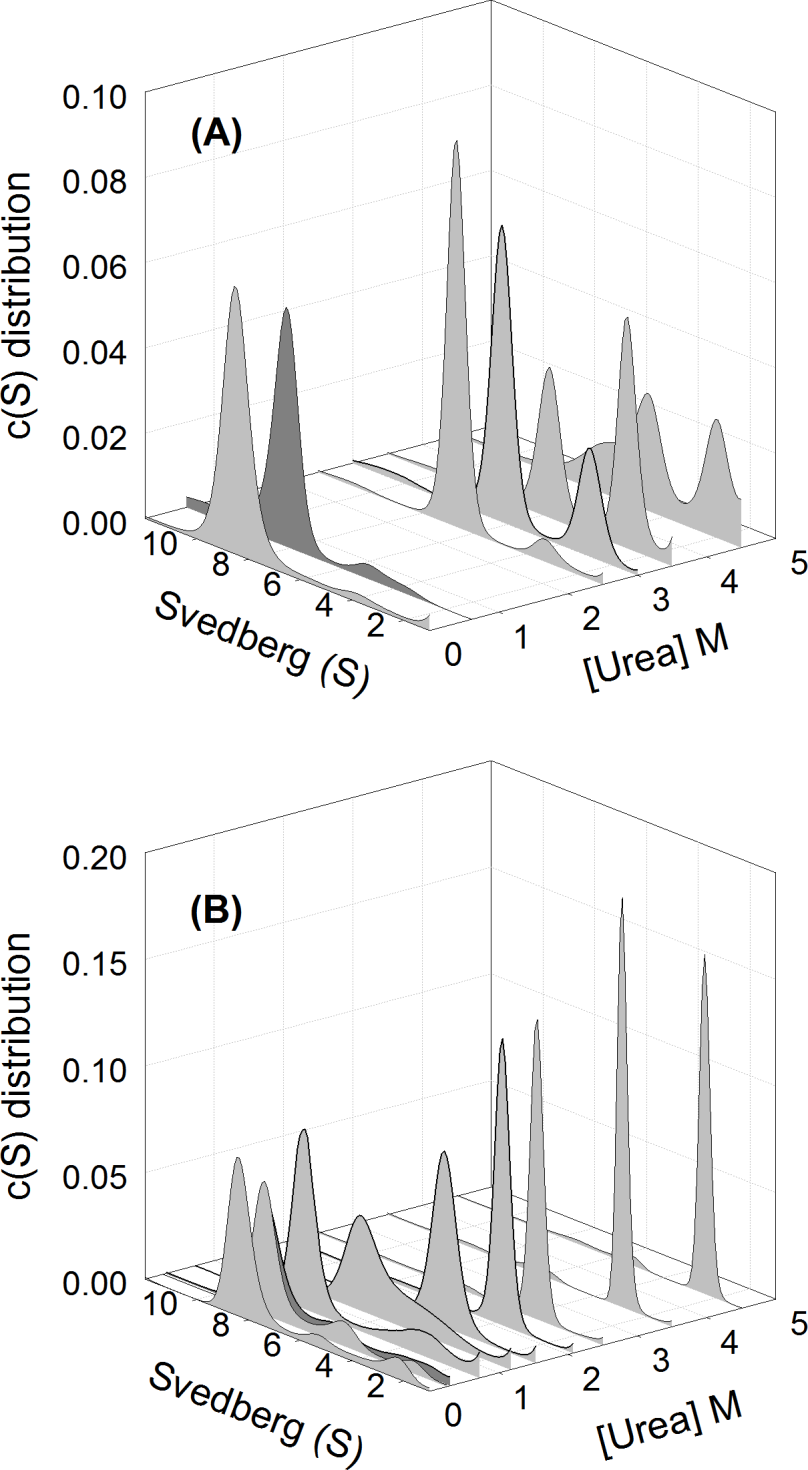
Sedimentation velocity analysis. The continuous sedimentation coefficient distribution of (A) wild-type and (B) K315A mutant protein at different urea concentration were shown. Peaks in grey color represent the unfolded wild-type and mutant protein in 0, 2.5, 3, 3.5 and 4.5 M urea and in 0, 0.25, 0.7, 1.3, 1.5, 2, 2.5, 3.5 and 4.5 M urea, respectively. Wild-type and mutant protein denatured in 3.5 and 2.5 M urea were diluted to final 0.6 and 0.3 M urea, respectively, in 50 mM Tris-acetic acid buffer, pH 7.5, were shown as dark gray peaks. The protein concentration used in all assays was 0.2 mg/mL.

The exposure of hydrophobic surfaces in the presence of urea was investigated using ANS titration. A dramatic increase in fluorescence was observed at around 3 M and 1 M urea, for wild-type and the K315A mutant, respectively, indicating that subunit dissociation had occurred due to the exposure of hydrophobic areas (Figs 2C and 3). The result is consistent with the observed changes in the tryptophan micro-environment as probed by tryptophan fluorescence (Fig 2B). The highest signals occurred around 2 and 5 M urea for the mutant and wild-type protein, respectively. Dilution of the 2.5 M urea denatured K315A mutant protein resulted in restoration of a native-like conformation. However, dilution of 4 or 6 M urea denatured wild-type or mutant protein, respectively, resulted in higher levels exposure of hydrophobic areas.

Since α-helices are the major secondary structure in δ-crystallin, the ellipticity at 222 nm was used to analyze the structural changes induced by the presence of urea (Fig 2B). The results showed both proteins retaining relatively stable α-helical structure at concentrations of urea below 2 M. There was about 13% and 30% loss of the structure at 3 M and 4 M urea, respectively.

### Effect of urea on the size-and-shape changes of δ-crystallin variants

**Fig 4:**
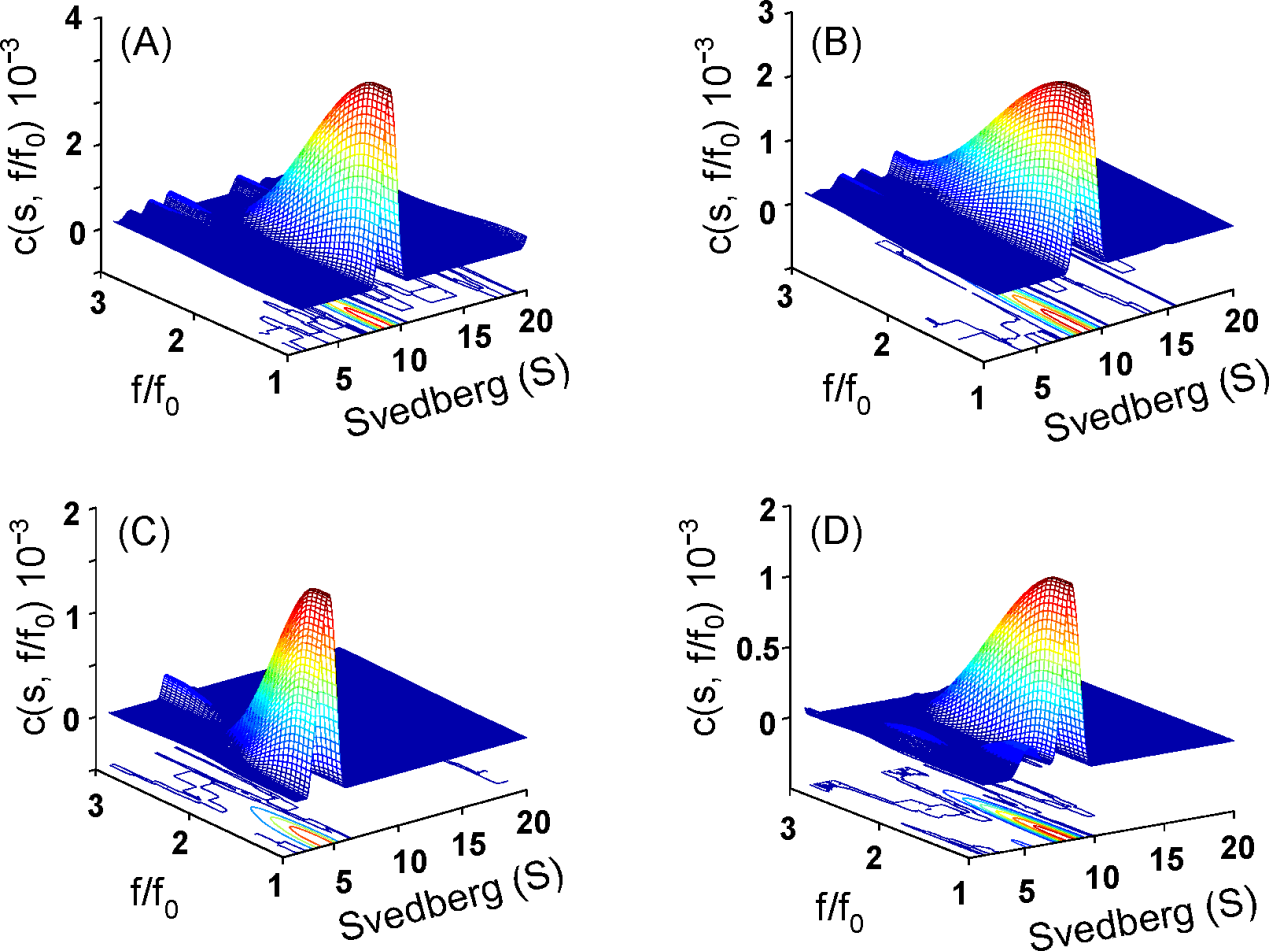
Size-and-shape distribution analysis. The sedimentation velocity experiments were performed and presented as the c(*s*, *f*/*f*_o_) distribution for (A) wild-type, (B) and (C) K315A protein in the absence and presence of 1.5 M urea, respectively. (D), K315A protein dissociated in 1.5 M urea was diluted to final of 0.26 M urea in 50 mM Tris-acetic acid buffer (pH 7.5).

The size and size-and-shape changes of wild-type and K315A mutant protein in different urea concentrations were determined by sedimentation velocity measurements and using continuous *c*(*s*) distribution and *c*(*s*, *f*/*f*_*0*_) distribution analysis, respectively (Figs 3 and 4) (S1 and S2 Figs) [29]. In the absence of urea, the two proteins appeared as one major component with sedimentation coefficients about 8.5 and 8.4 S, respectively (Fig 3). This peak corresponds to tetrameric δ-crystallin [11]. They possessed the native conformation as judged from the friction ratio (*f*/*f*_0_) distribution profile (with the centre region below 1.5 as shown by red in the contour) (Fig 4A and 4B).

At 2.5 M urea two components were observed for wild-type protein with sedimentation coefficients about 6.6 and 3.2 S, and these are assumed to be the dissociated dimeric and monomeric forms. The S values of the two peaks decreased with increasing urea concentration. The proportion of the second (monomeric) peak increased from 6% to 27% to 60% in the presence of 2.5, 3.0 to 3.5 M urea, respectively. Dilution of the denatured wild-type protein at 3.5 M urea resulted in refolding into one major component with an S value of 8.1 (Fig 3A). Measurement of ASL activity showed that around 25% activity was recovered following refolding (Table 1).

**Table 1:** Specific activity of δ-crystallin under the denaturant effect.

Renaturation conditions	Specific activity(nmol/min/mg)
Wild-type	
no treatment	4.4 ± 0.1
refolded from 3.5 M urea^a^	1.1 ± 0.2
refolded from 5 M GdmCl^b^	-^d^
K315A mutant	
no treatment	0.28 ± 0.03
refolded from 1.5 M urea	0.26 ± 0.1
refolded from 2.5 M urea	0.23 ± 0.08
refolded from 5 M GdmCl	-
refolded from 1.5 M urea and 5 M GdmCl^c^	0.9 ± 0.01
0.04 M urea and 0.13 M GdmCl	1.5 ± 0.05

^a^ The proteins unfolded in 3.5, 2.5 or 1.5 M urea were 20-fold diluted with 50 mM Tris-acetic acid buffer (pH 7.5).

^b^ The proteins unfolded in 5 M GdmCl were 40-fold diluted and incubated for overnight.

^c^ The mutant protein was unfolded in 1.5 M urea for overnight followed by addition of 5 M GdmCl and incubation for 2 hrs. Then, the protein was diluted 40-fold and incubated for overnight before activity measurement.

^d^ no detectable activity.

Subunit dissociation was observed at about 1.2 M urea for the K315A mutant protein, with S values for the major peak of 6.8 and a shoulder at about 4.5 S (Fig 3B). A single peak with an S value of 4.5 was observed for the mutant protein at ~1.5 M urea. This species is thought to be dissociated monomers possessing the native conformation as judged from the friction ratio (*f*/*f*_0_) distribution profile (Fig 4C). The monomers were reassembled into a similar quaternary structure of wild-type protein after removing the urea (Fig 4D). When the urea concentration was increased to 4.5 M, the S values for the single component were gradually decreased to about 2.4 (Fig 3B). Dilution of the protein denatured with 2.5 M urea resulted in refolding into one major peak with a S value of 8.1 S. The refolded protein showed around 80% ASL activity was recovered (Table 1).

### Conformational reversibility of monomeric K315A mutant δ-crystallin

**Fig 5:**
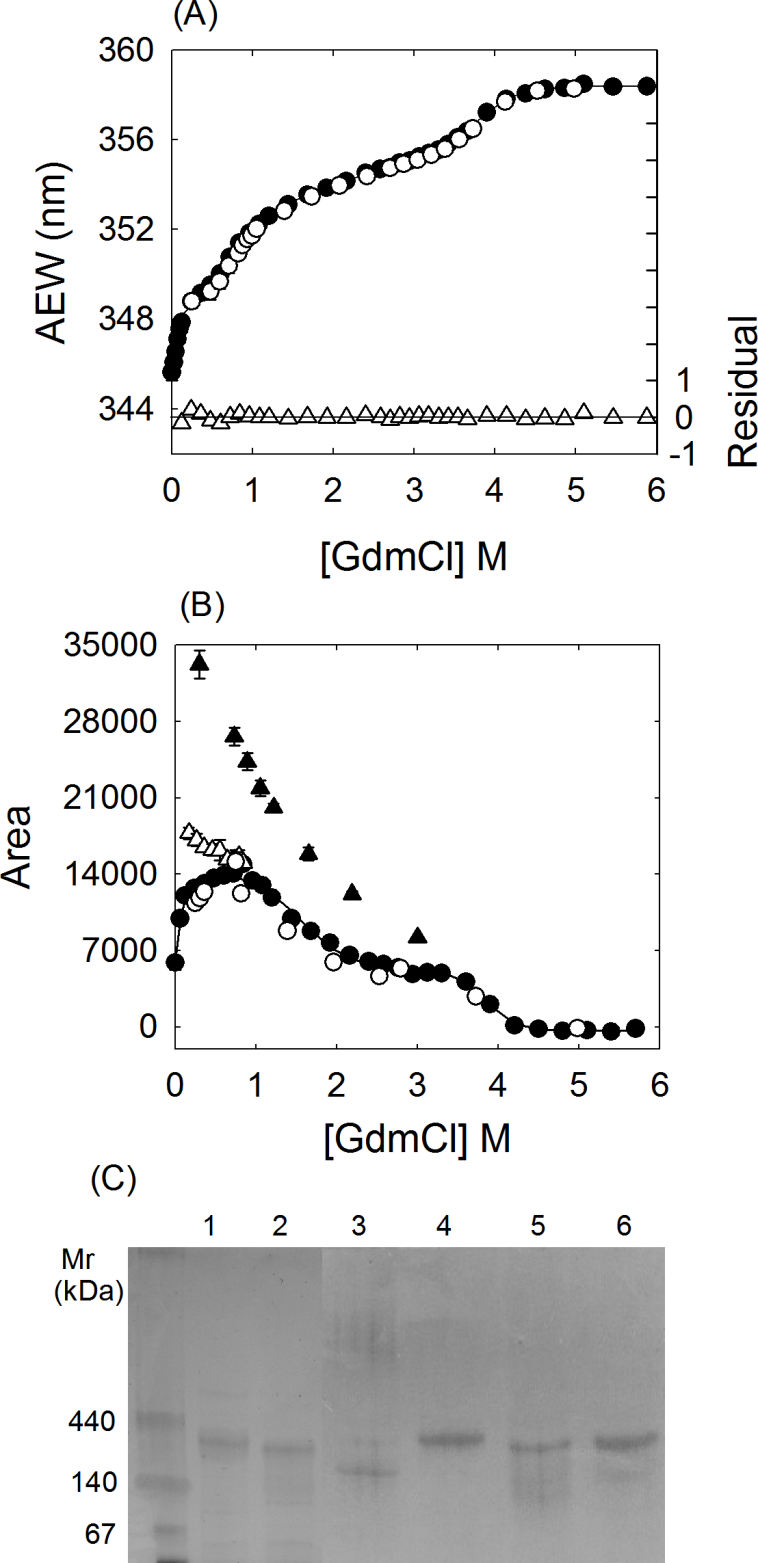
The unfolding and refolding of GdmCl denatured monomeric K315A mutant δ-crystallin. (A) Changes in average emission wavelength (AEW) of tryptophan fluorescence and (B) the integrated area of the fluorescence emission spectrum of ANS are presented as a function of GdmCl concentration. Monomeric K315A mutant δ-crystallin was incubated in 1.5 M urea before introducing varied GdmCl concentration. The labels of (●) and (○) represent the unfolding and refolding of monomeric K315A mutant protein, respectively. The (▲) and (∆) in (B) are the refolding transition from 3 and 0.8 M GdmCl denatured monomeric protein, respectively. The solid line in (A) is the best fitted result. The fitting residuals are shown as open triangles in (A). (C) The non-denaturing gel electrophoresis. Lanes 1 and 2 represent the wild-type and mutant protein without denaturant treatment, respectively. Samples from refolding of 5 M GdmCl denatured wild-type and mutant protein for time period of zero or overnight are shown in lanes 3–4 and 5–6, respectively. The protein concentrations used in the assays were 0.03, 0.1 and 0.1 mg/mL for (A), (B) and (C), respectively.

Since K315A mutant protein was reversible dissociated into stable monomers at 1.5 M urea, the conformational reversibility of monomers was investigated. Monomeric K315A mutant protein that had been produced by equilibrium incubation of the native protein in 1.5 M urea was treated with various GdmCl concentrations. Unfolding of monomeric K315A mutant proteins followed a multistep pathway as measured by both tryptophan and ANS fluorescence (Fig 5A and 5B). Stable intermediates were identified from the unfolding curve of ANS fluorescence which included the highest ANS fluorescence region at around 1 M GdmCl and a steady state at ~ 3 M GdmCl (Fig 5B). The monomeric protein had lost about 30 and 55% of its secondary structures at 1 and 3 M GdmCl, respectively. The changes in tryptophan fluorescence were correlated with the exposure of hydrophobic areas, which suggests the possible four-state unfolding model for monomeric δ-crystallin.

Dilution of monomeric K315A mutant protein denatured in 5 M GdmCl resulted in refolding to a similar conformation as the original monomeric state (Fig 5A and 5B). However, dilution of 1 and 3 M GdmCl denatured monomeric protein resulted in the increasing of the ANS fluorescence, indicating higher exposure of hydrophobic area (Fig 5B). The results suggest the conformation of the partly unfolded intermediate could affect the folding reversibility of the monomeric K315A δ-crystallin mutant.

### Thermodynamic parameters calculation

K315A mutant δ-crystallin that denatured in 3 M urea was reversible folded back to the original conformation after dilution (Fig 2B). Signal changes in the tryptophan fluorescence with different urea concentration were used to calculate the thermodynamic parameters by directly fitted to the two-state mechanism (Eq 1) [19]. The free energy difference in the absence urea (ΔG^0^) for the transition was determined to be 6.5 ± 0.3 kcal/mol (Table 2).

**Table 2:** Thermodynamic parameters for K315A mutant δ-crystallin.

Equilibrium unfolding	Δ*G*_u_^0^ (kcal/mol)	*m* (kcal/mol/M)	[D]_1/2_ (M)
T ↔ M^a^	6.5 ± 0.3	3.8 ± 0.2	1.7 ± 0.05
M↔I_1_^b^	1.3 ± 0.2	2.1 ± 0.4	0.6 ± 0.04
I_1_↔I_2_	2.6 ± 0.8	1.2 ± 0.2	2.1 ± 0.2
I_2_↔U	8.9 ± 0.4	2.3 ± 0.1	3.9 ± 0.1

^a^ The reversible dissociation process was described as a two-state transition from the conversion of the tetramer (T) to monomeric intermediate (M). [D]_1/2_ is the concentration of denaturant at which the transition is half completed. The data was calculated by global fitting to Eq 1.

^b^ The data were fitted to a 4-state unfolding model (Eq 2) described as the transition from M to U. Two intermediates, I_1_ and I_2_, were assumed in the process before denatured species (U). These data are the mean ± SD of at least three independent experiments.

The changes of tryptophan fluorescence as a function of GdmCl concentration were used to calculate the thermodynamic parameters for the reversible unfolding of the monomeric K315A δ-crystallin mutant (Fig 5A). The unfolding curve was best fitted into a four-state model [8,20]. The [GdmCl]_1/2_ for the transitions from the M to I_1_, I_1_ to I_2_ and I_2_ to U (denaturation) were about 0.6 ± 0.04 M, 2.1 ± 0.2 M and 3.9 ± 0.1 M, respectively. The thermodynamic parameters determined are summarized in Table 2. The total free energy difference (ΔG^0^) for folding of monomeric K315A δ-crystallin mutant was determined to be 12.8 ± 0.7 kcal/mol.

### Reversibility of the quaternary structure from denatured monomeric K315A mutant δ-crystallin

To determine the ability about refolding of denatured monomeric K315A mutant followed by reassembly to a tetrameric protein, the monomeric proteins that denatured by GdmCl were diluted with buffer to remove both of the urea and GdmCl. The results showed that the quaternary structure of K315A mutant protein was recovered from the denatured monomers instantly after dilution. The amount of the assembled protein was increased with the incubation time possessing about 60% of the activity recovered (Fig 5C and Table 1). In contrast, dilution of 5 M GdmCl denatured wild-type δ-crystallin, the quaternary structure was recovered after overnight incubation with no detectable activity (Table 1). The similar result was also shown for 5 M GdmCl denatured K315A mutant protein. These results suggested the different pathway for protein folding that seems due to the distinct conformation of the denatured protein caused by different means of denaturation.

### Protein aggregate formation from refolding of monomeric intermediate

**Fig 6:**
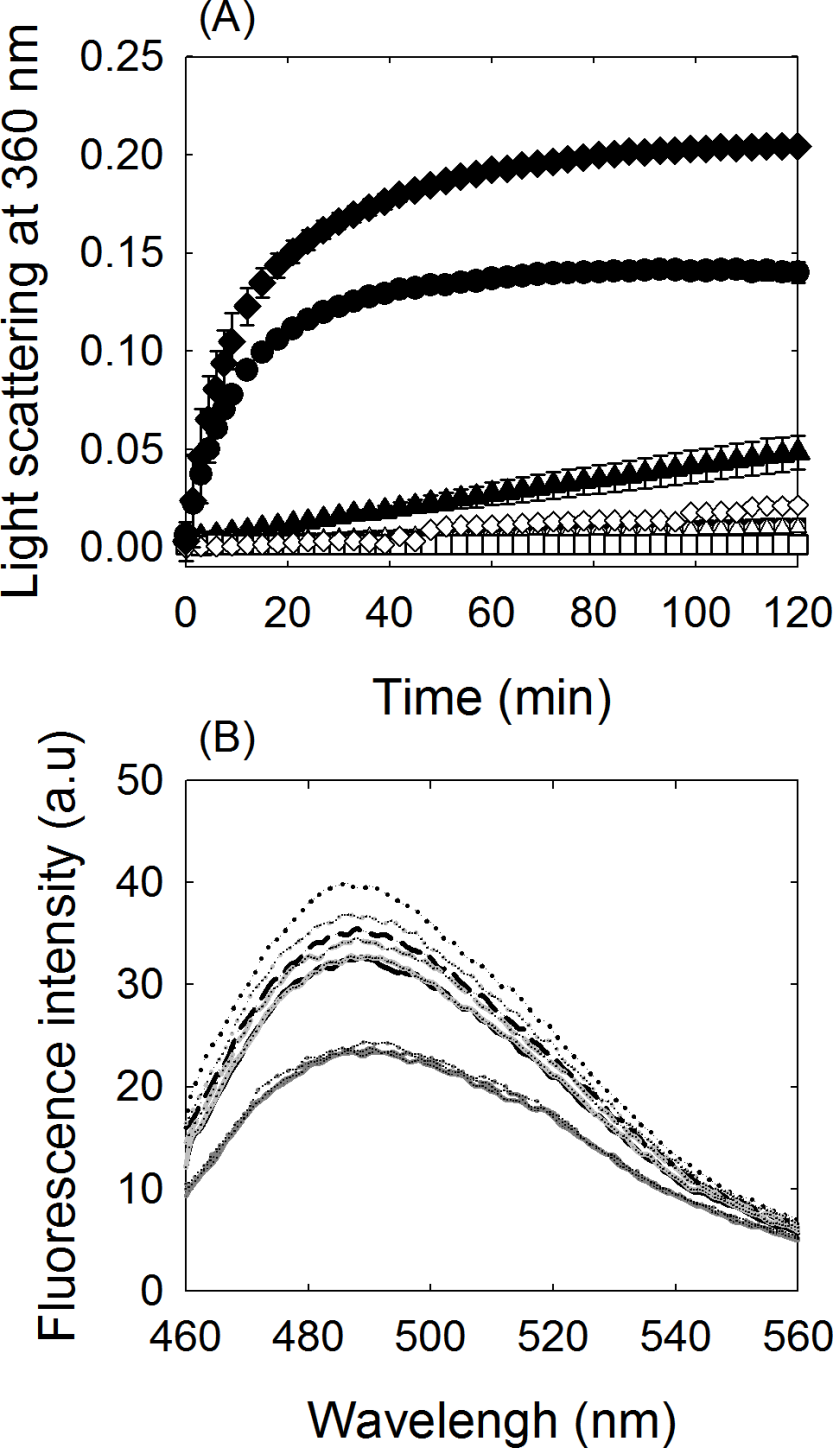
Aggregates formation from refolding of denatured monomeric K315A mutant δ-crystallin. (A) Light scattering measurement. Monomeric K315A mutant δ-crystallin was incubated in 1.5 M urea before introducing varied GdmCl concentration. Refolding of 0.84, 3 and 5 M GdmCl denatured monomeric K315A mutant protein by dilution with 50 mM Tris-acetic acid buffer (pH7.5) or the buffer containing 1.5 M urea are shown as (●, ▲ and ■) or (○, ∆, and □), respectively. The label of (♦) represents refolding of 0.84 M GdmCl denatured monomers by dilution with buffer including αA-crystallin. αA-Crystallin alone in 1.5 M urea and 0.84 M GdmCl is shown as (◊). The protein concentrations of monomeric K315A and αA-crystallin used in the assays were 0.1 and 0.5 mg/mL, respectively. (B) The fluorescence spectrum of ThT were shown from refolding of 0.84 (dark), 3 (gray) and 5 M (dark gray) GdmCl denatured monomeric K315A mutant protein by dilution with buffer and incubated for zero (solid-line), 1 (dash-line) or 2 (dot-line) hrs, respectively.

Since refolding of partly unfolded monomeric mutant δ-crystallin resulted in a conformation with high exposure of hydrophobic regions, the occurrence of protein aggregation in the process was determined using light scattering measurement. No protein aggregation was detected upon dilution of 0.84, 3 and 5 M GdmCl denatured monomeric mutant protein into buffer containing 1.5 M urea. However, when 0.84 and 3 M GdmCl denatured monomeric mutant protein was diluted into buffer, protein aggregation was occurred. The rates for aggregate formation were calculated to be *ca*. 0.14 and 0.0004 min^-1^, respectively (Fig 6A). When αA-crystallin, the chaperone protein, was added in a 5:1 ratio to 0.84 M GdmCl denatured monomeric mutant protein in folding buffer, no change in the rate of protein aggregation was observed. Formation of aggregates by αA-crystallin alone did not occur under the same conditions. It is notable that upon dilution of 5 M GdmCl denatured monomeric mutant protein into buffer, no aggregation occurred.

The structural features of the protein aggregate were investigated using the thioflavin T assay [30]. An increase in fluorescence intensity resulting from binding of ThT with the aggregates over time was observed following dilution of 0.84 and 3 M GdmCl denatured monomeric mutant δ-crystallin into buffer (Fig 6B). The results suggest the possible formation of ordered aggregates. No changes in the signal were observed during the incubation period upon refolding of 5 M GdmCl denatured monomeric mutant protein.

### Molecular dynamics simulation

**Fig 7:**
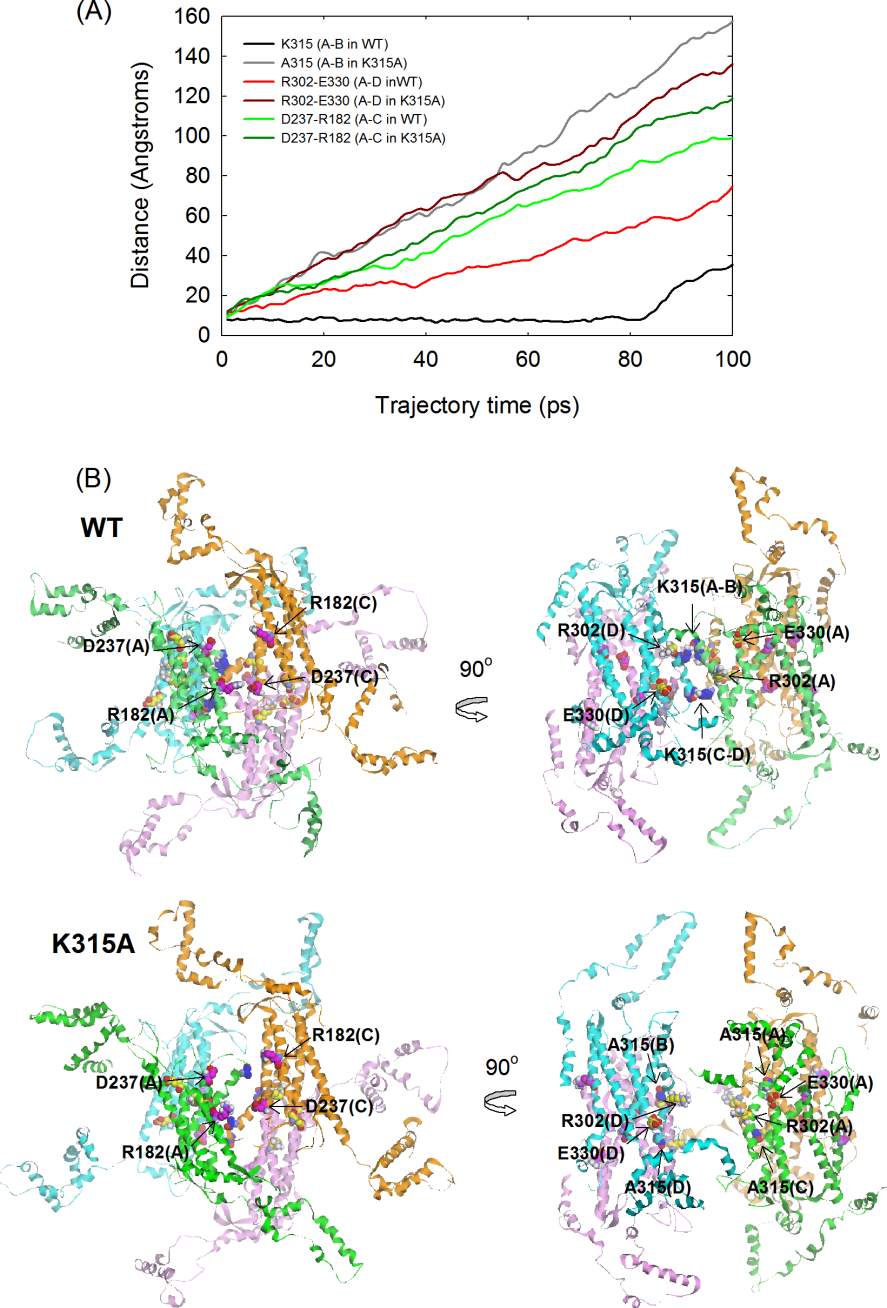
Molecular dynamics simulations. (A) The distances between the C_α_ of specific residues located at the interfaces along the simulation. (B) The conformation of wild-type and K315A mutant protein at trajectory frame of 20 ps.

To determine the effect of the interactions provided by K315 at the diagonal subunits in disassembly of the quaternary structure, a MD simulation were run for 100 ps for wild-type and mutant δ-crystallin in the absence of any structural restraints. From the simulation trajectory, the dynamic motion for disassembly of the quaternary structure and conformational changes in the tertiary structure were elucidated. The distances between the C_α_ of D237 and R182, R302 and E330 and the two K315 or A315 residues were measured to evaluate the extent for subunit dissociation between the A-C, A-D and A-B dimeric pairs, respectively (Fig 7A). These residues interact with each other by hydrogen bonding or salt bridges at the dimeric pair interface in the native structure. These interactions are lost on replacement of K315 with A315. The results showed that the distances between D237 and R182, R302 and E330 and the two A315 residues increased linearly at a similar rate, except that the rate of change for the R302-E330 interaction in wild-type protein was about half of that for the mutant protein. In contrast, no changes in the distance between the K315 residues were observed before 80 ps. Inspection of the time-course at 20 ps in wild-type protein showed that the primary dimers of subunit A and C or B and D showed were separated, while the diagonal dimers of subunit A and B or C and D were connected by the interactions of residue K315 (Fig 7B). However, the subunits for both of the primary and diagonal dimers were separated from each other in the mutant protein. The results suggest a different disassembly process for tetrameric wild-type and mutant proteins.

## Discussion

The quaternary structure of δ-crystallin is assembled as two pairs of closely associated dimers. Previous mutagenesis studies have used to investigate the interactions at the interfaces of double dimers to elucidate their role in the stabilization of the quaternary structure [11]. The unique stable conformation from unfolding of K315A mutant protein in the presence of urea suggests that the interactions provided by this residue at the interfaces may play a critical role in stabilization of the quaternary structure of δ-crystallin.

Lys-315 is the only residue which is arranged symmetrically at the diagonal subunit interfaces (Fig 1B). The ε-amino group in the side-chain of this residue forms hydrogen bonds with the carbonyl groups of M312, V313 and K315 within the symmetric subunit (from PISA analysis: http://www.ebi.ac.uk/msd-srv/prpt_int/cgi-bin/piserver) (Fig 1D). Substitution of this residue by alanine reduces the structural stability of the protein. The results from the previous study showed about a 9°C reduction in the thermal stability of the secondary structure and the changes in the micro-environment surrounding the tryptophan residues [11]. This mutant protein was also more susceptible to chemical denaturation, since about half of the concentration of denaturant was required to disrupt its quaternary structure compared to wild-type protein. Both of the wild-type and mutant protein showed similar and not reversible denaturation in the presence of GdmCl. However, differences in the denaturation pathway were observed when urea was used as the denaturant. The results suggest that the non-covalent interactions between the intra and inter-subunits might be disrupted by the ionic character of GdmCl, with subunit dissociation and denaturation occurring simultaneously for both proteins [31]. The previous studies showing the presence of only a monomeric molten globule intermediate in the dissociation pathway of wild-type δ-crystallin in GdmCl supports this assumption [12,13]. Thus, the role of K315 in the folding process of δ-crystallin cannot be distinguished under the strong denaturant.

Around 2~5 M urea, the K315A mutant δ-crystallin is in a stable conformation, as judged by tryptophan fluorescence measurements (Fig 2B). Subunit dissociation occurs under these conditions, resulting in the exposure of hydrophobic regions (Figs 2C and 3B). Only monomers were identified in this state, as measured by sedimentation velocity analysis. The monomers that dissociated from the tetramers at ~1.5 M urea possessed a nearly identical content of secondary structure as native protein and native-like conformation (Figs 2B and 4C). Monomers in this conformation were able to refold and re-associate into tetramers with a similar conformation as the native protein, and possessed significant ASL activity. When the urea concentration was increased to 2.5 M, the conformational changes led to further exposure of hydrophobic regions. However, following this conformational change at higher urea concentrations, protein folding was not reversible. The contrasting result found for wild-type protein was that the dissociation and denaturation were concurrent under the effect of urea (Fig 3A). In this condition, the conformation of the dissociated monomers was partly unfolded, as judged from the reduced level of α-helix (Fig 2B). The dissociated monomers seem to refold into alternative conformations then re-associate into tetramers with only part of the catalytic activity recovered (Table 1). These results indicated that the conformation of the monomers seems related to the assembly pattern for functional protein.

**Fig 8:**
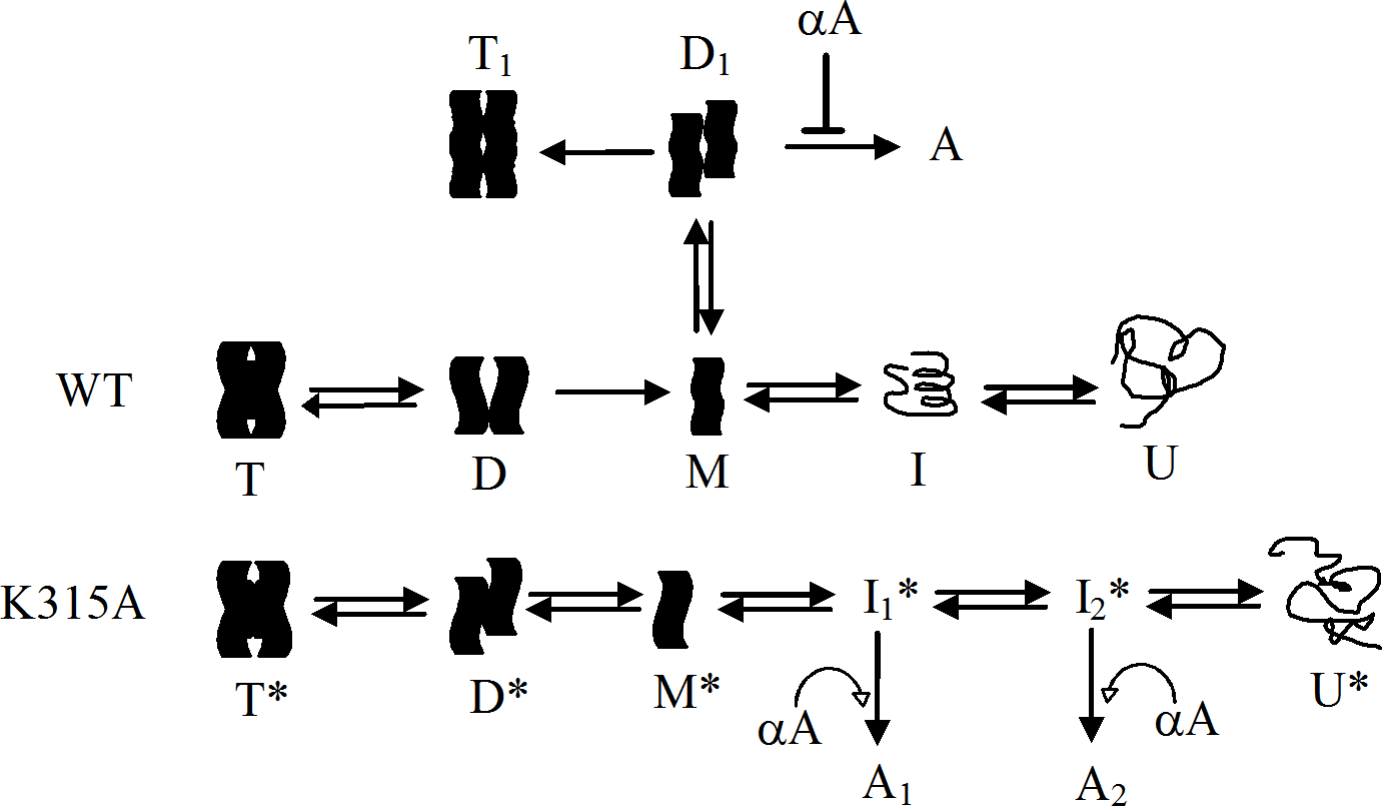
A working model proposed for the folding pathway of wild-type and K315A mutant δ-crystallin. The tetrameric wild-type (T) and K315A mutant (T*) δ-crystallin was dissociated through the diagonal dimer (D) and primary dimer (D*) to monomers with partial unfolded (M) and stable (M*) conformation, respectively. Monomers of the wild-type or the mutant protein were then denatured through intermediate (I or I*) into respective unfolded form (U or U*). The monomers (M) of wild-type protein in partial unfolded conformation was associated in alternative pathway to form dimers (D_1_), and then assembled into tetramers (T_1_) or aggregates (A). The aggregation was prevented by αA-crystallin. Refolding followed by assembly of the intermediates (I_1_* and I_2_*) of the mutant protein resulted in the aggregates (A_1_ and A_2_) formation and the chaperone function of αA-crystallin was invalid in this process.

It is interesting that the interactions provided by K315 at the interfaces seem to affect the disassembly pathway of the quaternary structure of wild-type protein. It was found that both of the dimers and monomers were dissociated from the wild-type protein at around 2 to 4 M urea, as measured by sedimentation velocity while only monomers were dissociated from the mutant protein (Fig 3). These results suggest that the interactions provided by K315 at the interfaces seem to increase the energy barrier for dimeric dissociation. When these interactions were disrupted by mutation, the monomers could be isolated with intact conformation from the tetramers earlier than for the wild-type protein in urea. Thus, the steps for dissociation and denaturation could be distinguished in the unfolding process of the K315A mutant δ-crystallin. In this study, the dynamic motion of protein structure in the process was simulated for elucidation the dissociation mechanism of δ-crystallin. From the trajectory, the tetramers were found to disassemble at the early stage. Due to the interactions by K315, the interfaces between the diagonal dimers remain connected while distances increase between the subunits of the primary dimers in wild-type protein. In contrast, the subunits between both of the diagonal and primary dimers were dissociated in the K315A mutant protein (Fig 7). As δ-crystallin was assembled by two close contact dimers as transthyretin, dissociation at the two primary dimer interfaces would be expected to occur at the initial stage [4,32]. The simulation result provides a novel pattern for dissociation of the double dimeric protein consistent with the results of sedimentation velocity experiments. A possible explanation for this earlier dissociation of the subunit from the primary dimers compared to diagonal dimers is differences in solvent accessibility. Unlike the location of the interfaces between the subunits of the primary dimer, the position of K315 is buried at the interior interfaces away from solvent. Thus, the interactions of K315 at the interfaces of the protein seem to elevate the stability of the quaternary structure. For wild-type protein, two diagonal dimers were presumed to disassemble initially from the tetramers followed by subunit dissociation of the diagonal dimers. However, dissociation at the interfaces of two primary dimers would assume to be the first step in the unfolding process of the K315A mutant protein (Fig 8).

The detail mechanism for folding of monomeric protein remains elusive due to the monomers dissociated from wild-type δ-crystallin were in a molten globule conformation. Thus, the monomers that reversible dissociated from K315A mutant δ-crystallin with a stable conformation and possessing similar level of secondary structure as the original state, and this would be a good model for studying the folding process. The monomeric protein was reversible denatured in a four-state mechanism in the presence of GdmCl and two intermediates were detected in the process (Fig 5). Refolding of the partly unfolded intermediate was not reversible which in turn resulted in a conformation with more exposure of hydrophobic regions. Only denatured δ-crystallin was reversible folded into the monomers with a similar conformation to the original state. It is interesting that the refolded monomers were able to reassemble into tetramer instantly upon dilutions, with substantial recovery of activity (Fig 5 and Table 1). This contrasts with the slow refolding of GdmCl denatured wild-type protein into its tetrameric form with no detectable activity. The slow recovery of the quaternary structure for the latter protein is due to an energy barrier for the appropriate assembly of double dimers, as reported previously [14]. The results suggest that the conformation of the denatured monomers which was unfolded by stepwise dissociation or directly unfolded with 5 M GdmCl could be different. The consequence of this might be that protein folding occurs *via* different pathways leading to the refolded monomers with different conformations to associate into native structure or alternative structures without function.

Protein aggregates are prone to form during the reassembly process from refolding of partly unfolded monomeric intermediates of δ-crystallin. The intermediate with the highest exposure of hydrophobic conformation is particularly prone to aggregate formation (Fig 5). Aggregate formation by monomeric intermediates with defined conformations was also reported for transthyretin under mildly acidic conditions [33]. The result implies that the conformational status of the monomers influences subunit association. It is interesting that the presence of α-crystallin seems to increase the formation of aggregates from the monomeric intermediates of δ-crystallin with partly unfolded conformation, while α-crystallin alone was not affected under these conditions (Fig 6). The studies for αA peptide which induces the aggregation of soluble α-crystallin suggested that the mechanism for aggregate formation might due to the changes in the hydrophobicity of α-crystallin induced by the peptide [34,35]. Our previous study reported that aggregate formation during refolding of GdmCl denatured wild-type δ-crystallin was due to the improper assembling of double dimers and was prevented by the presence of α-crystallin [14]. In this study, the aggregate formation was caused by assembly of the refolded monomeric intermediate which facilitated the aggregate formation of α-crystallin. It thus postulated that the electrostatic interaction with the substrate seems to be key factors to determine the chaperon-like or anti-chaperone activity of the α-crystallin [34,36]. The underlying mechanism requires further investigation. Nonetheless, the result highlights the conformational status of the monomers which play a critical role in the folding pathway for reversible oligomerization or aggregate formation.

In conclusion, the folding pathways of wild-type and mutant δ-crystallin are summarized as the working models shown in Fig 8. This model depicts the key interactions from K315 at the interfaces of diagonal subunits not only to stabilize the quaternary structure of δ-crystallin but also to act as the energy barrier for dissociation of stable monomers. The stability might be one of the reasons for recruitment of the metabolic enzyme ASL into the lens as a crystallin protein [37]. The single polypeptide chain of δ-crystallin after translation would be assumed to fold into functional tetramers as the proposed refolding pathway for K315A mutant. However, due to the interactions by K315, the tetrameric protein would be assumed to dissemble in an alternative manner to form the diagonal dimers, followed by simultaneous subunit dissociation and denaturation. Monomers in this status might associate into dimers *via* a different pathway which then assemble slowly into a non-native tetrameric form or self-associate into aggregates which can be prevented by the presence of α-crystallin. The reversible folding of the monomers that dissociated from the K315A mutant protein with near native conformation provided the folding mechanism of the δ-crystallin. In this process, the ordered aggregate formation from re-association of the partly unfolded intermediate reveals a specific status of the protein to avoid the chaperone function of α-crystallin. This model proposes a possible mechanism about the aggregate formation for lens protein under stress effect and their interaction with α-crystallin. This study reveals the key role of monomers that dissociated from the oligomeric crystallin; their conformational status determines the levels of aggregate formation.

## Supporting Information

S1 Fig Sedimentation velocity analysis of wild-type δ-crystallin. (A) and (B), the panels show the raw sedimentation and theoretical fitted data (solid lines), and the fitting residual, respectively. (C) Grayscale of residual bitmap. The raw sedimentation data were fitted to the continuous size distribution model including the solvent using the SEDFIT program [25].
(TIF)

S2 Fig Sedimentation velocity analysis of K315A mutant δ-crystallin. (A) and (B), the panels show the raw sedimentation and theoretical fitted data (solid lines), and the fitting residual, respectively. (C) Grayscale of residual bitmap. The raw sedimentation data were fitted to the continuous size distribution model using the SEDFIT program [25].
(TIF)

## Abbreviations

ANS: 1-anilinonaphthalene-8-sulfonic acid
GdmCl: guanidinium hydrochloride
CD: circular dichroism
AEW: average emission wavelength
λ_max_: maximum emission wavelength
C_m_: half concentration of denaturant required in protein unfolding
MD: molecular dynamics
