# Supplementary figures and images for: Lys-315 at the Interfaces of Diagonal Subunits of δ-Crystallin Plays a Critical Role in the Reversibility of Folding and Subunit Assembly
Source: PLoS One. 2016 Jan 5;11(1):e0145957. doi: 10.1371/journal.pone.0145957 (PMC4701392; doi:10.1371/journal.pone.0145957)

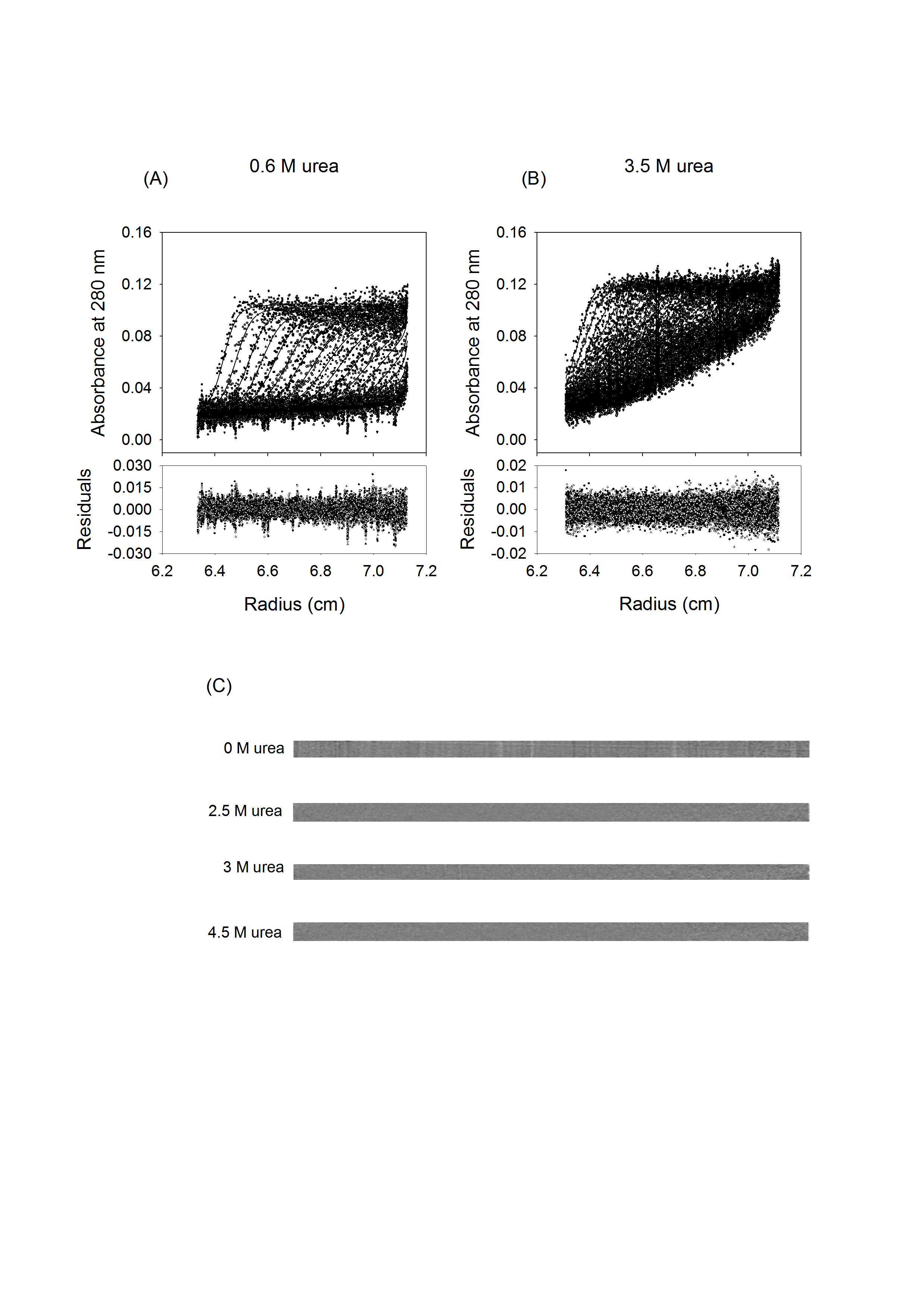

Supplement: S1 Fig — (A) and (B), the panels show the raw sedimentation and theoretical fitted data (solid lines), and the fitting residual, respectively. (C) Grayscale of residual bitmap. The raw sedimentation data were fitted to the continuous size distribution model including the solvent using the SEDFIT program [25]. (TIF) [file pone.0145957.s001.TIF]

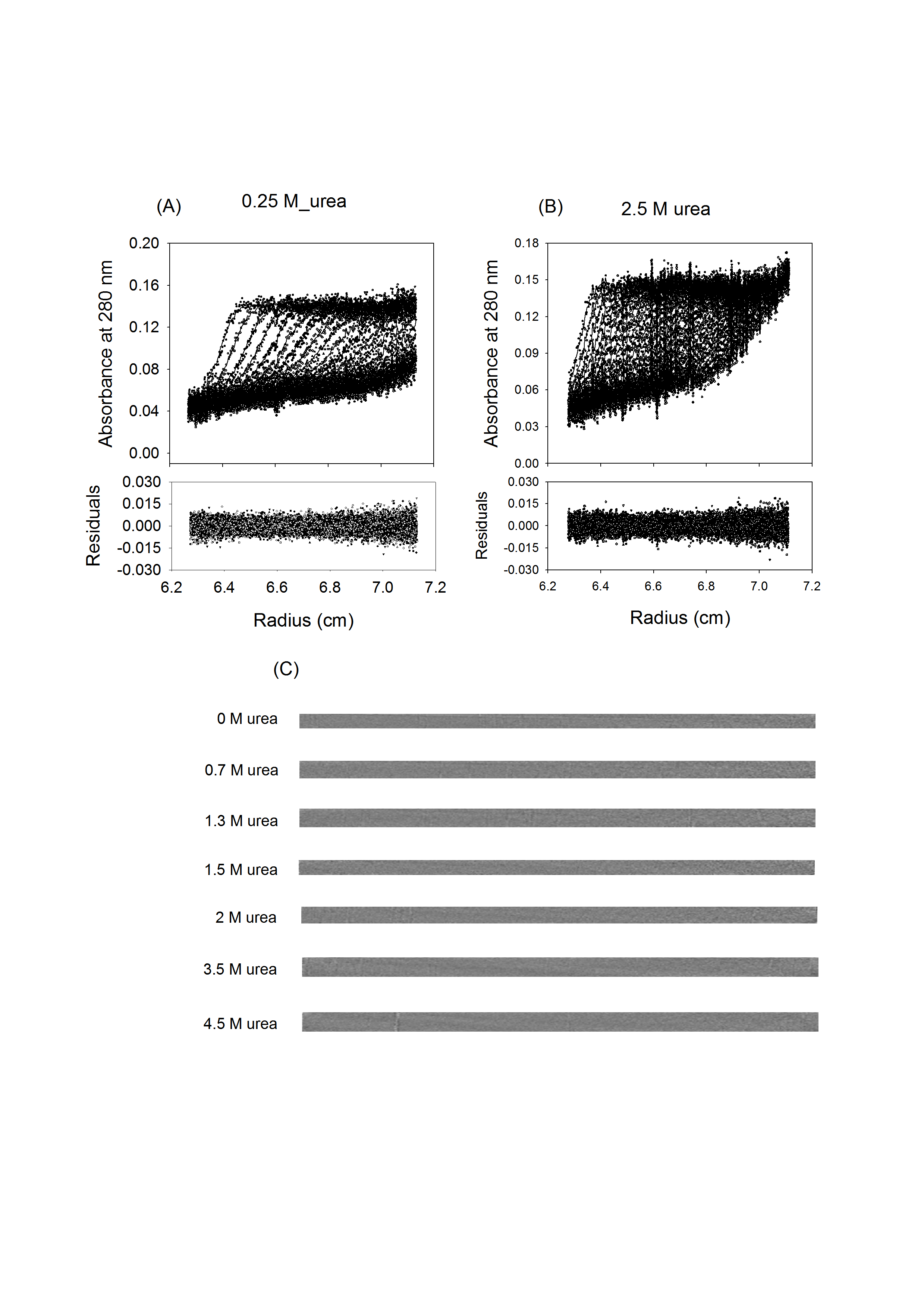

Supplement: S2 Fig — (A) and (B), the panels show the raw sedimentation and theoretical fitted data (solid lines), and the fitting residual, respectively. (C) Grayscale of residual bitmap. The raw sedimentation data were fitted to the continuous size distribution model using the SEDFIT program [25]. (TIF) [file pone.0145957.s002.TIF]
